# Towards autonomous energy management: machine learning for effective auditing and optimization

**DOI:** 10.1038/s41598-025-24513-7

**Published:** 2025-11-10

**Authors:** Sherif Ashraf, Mira M. Zarie, Sameh O. Abdellatif

**Affiliations:** The Electrical Engineering Department and FabLab, Centre of Emerging Learning Technologies CELT, British University in Egypt (BUE), Cairo, 11387 Egypt

**Keywords:** Energy management, Machine learning, Load classification, Energy savings, Sustainability, Energy science and technology, Engineering, Mathematics and computing

## Abstract

This study presents a fully automated procedure for energy management and auditing, applicable to a diverse range of residential and commercial loads, leveraging machine learning techniques across three key phases: load classification, benchmarking, and smart monitoring. The model effectively categorizes energy loads based on consumption patterns, establishes performance benchmarks through historical data analysis, and employs real-time monitoring to identify inefficiencies and predict future energy usage. Evaluating the model through four distinct case studies demonstrates its capability to optimize energy consumption in a techno-economic manner, achieving significant energy savings of 34.73 MWh/year for essential loads in Egypt, 215.67 MWh/year for HVAC systems in a university building, 0.9 MWh/year for a hybrid lighting system in a bank branch, and 0.9 MWh/year for a residential house. The results underscore the model’s effectiveness in promoting energy efficiency and sustainability, highlighting its transformative potential in adapting to the evolving energy needs of various applications while facilitating substantial cost savings.

**Supplementary Information:**

The online version contains supplementary material available at 10.1038/s41598-025-24513-7.

## Introduction

The industrial sector is responsible for a significant portion of global energy consumption, with inefficiencies in energy usage contributing to increased operational costs and environmental concerns^1–3^. Energy management involves monitoring system energy use to enhance performance and promote environmental sustainability in industrial facilities^4^, as well as integrating renewable energy supplies^5–7^. Previous efforts to address these inefficiencies primarily relied on traditional energy management systems, which, despite being widely adopted, often fall short in adapting to real-time changes in energy demand or operational conditions^4,8–13^. Early research focused on demand-side management and load-scheduling strategies. While these methods provided some improvements, their ability to predict and respond to dynamic energy needs remained limited, highlighting the need for more adaptive approaches.

In recent years, the application of Artificial Intelligence (AI) has emerged as a promising solution for optimizing energy management. This research adopts AI-driven strategies, particularly supervised machine learning (ML) using tools from the Python framework, to improve industrial energy systems^9,11,12,14–18^. It prioritizes sustainable lighting and business data management strategies, particularly through ML techniques that enable the analysis of large datasets to uncover patterns in energy consumption and predict future usage^19–23^. Studies have demonstrated the potential of machine learning models, such as Support Vector Machines (SVM)^24^ and decision trees^25^, in improving the accuracy of energy consumption forecasts. For example, L. A. Yousef, et al.^26^, applied SVM to predict energy consumption in industrial settings, achieving more accurate energy-saving adjustments.

Despite the promise of AI in energy management, its integration into existing systems has posed several challenges. Researchers like M. Grunt et al.^27^ have identified difficulties in merging AI with legacy infrastructures, as this requires significant adjustments to existing processes. Additionally, the performance of machine learning models can be heavily influenced by the quality and consistency of data. As Mortaj et al. pointed out, incomplete or unreliable data can compromise the predictions made by AI models, limiting their practical application^28^. Load optimization enhances energy efficiency by reducing power spikes and preventing unnecessary energy consumption while maintaining system stability^29^. This strategy must overcome technological barriers and faulty records by employing AI systems to balance loads in real-time, utilizing IoT updates to audit energy use, and implementing scalable business knowledge systems to minimize expenses and support sustainable practices^30^.

Considering these developments, AI techniques, particularly supervised machine learning using Python applications, offer significant potential for enhancing energy efficiency in industrial settings^1,9,30^. The extensive libraries available in Python simplify AI model development tasks, making it a preferred choice for industrial AI applications due to its flexibility and organized community-based support systems. With the continued evolution of AI models, alongside advancements in data processing and integration with existing infrastructure, AI is expected to play a crucial role in overcoming remaining challenges. With further research and development, AI is anticipated to significantly optimize energy consumption, contributing to sustainability and operational efficiency in the industrial sector. Research indicates that AI optimization systems can conserve up to 20% of industrial energy while reducing operational costs and environmental impact. Numerous studies show that AI-based preventive maintenance can decrease downtime by an impressive 30%, thereby increasing production output. Herein, while screening the literature, we recognized a research gap in integrating AI-based solutions in energy management techniques with full utilization and automation.

**Fig. 1:**
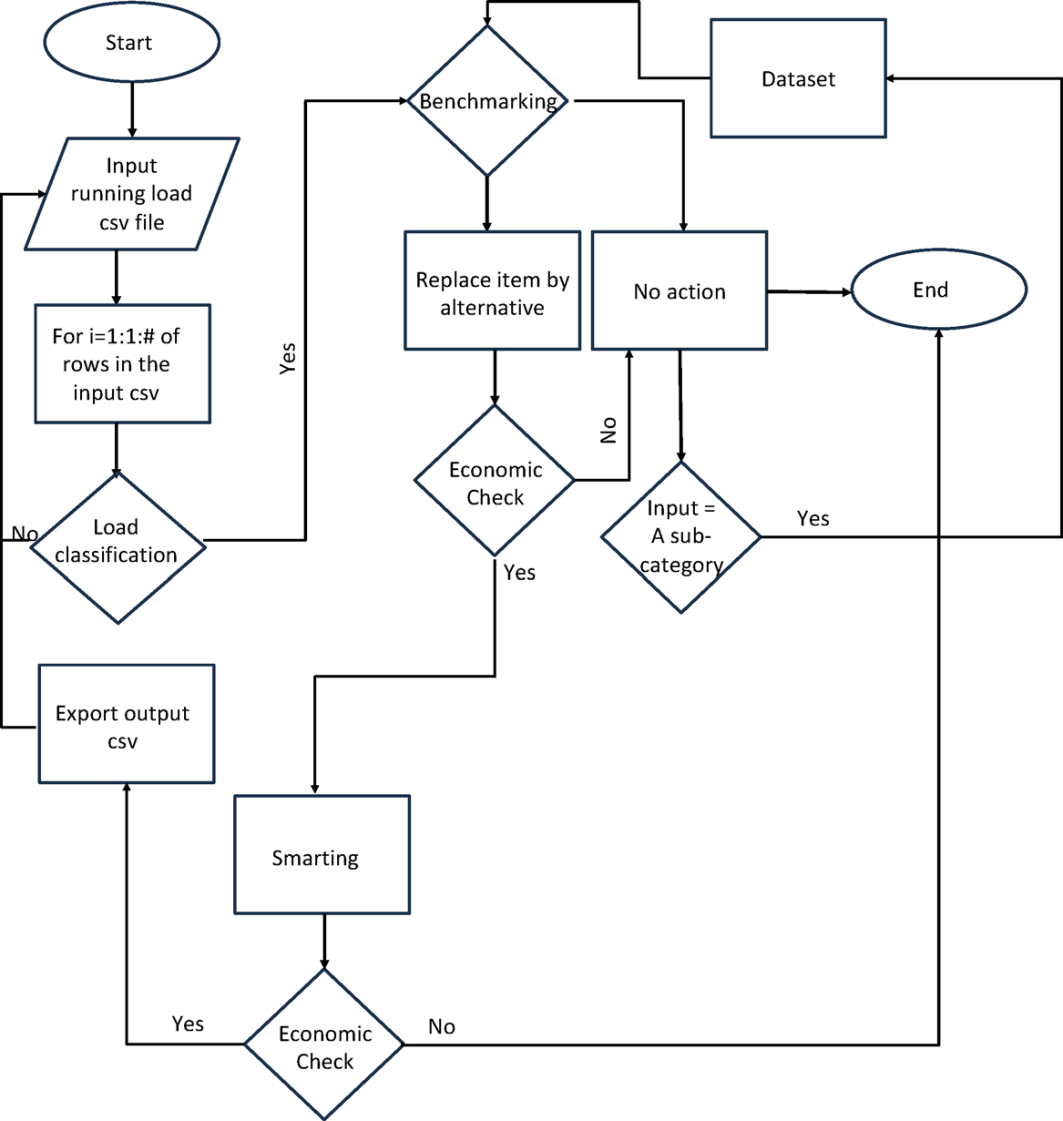
A flow-chart demonstrating the entire AI-based energy management model.

The current study presents a comprehensive and fully automated procedure for energy management and auditing applicable to a variety of residential and commercial loads. This innovative model leverages machine learning techniques and is implemented in three main phases: load classification, benchmarking, and smart monitoring. Following these phases, energy management calculations are conducted to evaluate the model’s outputs relative to the input conditions (see the flowchart in Fig. 1). In the load classification phase, the model accurately categorizes different types of energy loads based on their consumption patterns and operational characteristics. This classification is essential for tailoring energy management strategies to the specific needs of each load type, whether residential appliances, commercial equipment, or industrial machinery. The benchmarking phase involves establishing performance standards against which the energy consumption of the classified loads can be compared. By analyzing historical data and industry standards, the model identifies optimal energy usage benchmarks, enabling users to understand how their current energy consumption aligns with best practices.

In the smart monitoring phase, real-time data is collected and analyzed to track energy usage continuously. This allows for the identification of inefficiencies and anomalies in energy consumption, providing insights that can lead to immediate corrective actions. Machine learning algorithms enhance this phase by predicting future energy consumption patterns based on historical data, enabling proactive management of energy resources. Subsequently, energy management calculations are performed to assess the model’s outputs in relation to the current input status. This evaluation provides a clear picture of energy savings achieved and identifies areas for further improvement. The robustness of the model is verified through a series of case studies, which demonstrate quantifiable energy savings measured in kilowatt-hours (kWh). These case studies not only validate the effectiveness of the automated energy management and auditing procedure but also highlight its applicability across various settings. The results indicate significant reductions in energy consumption, showcasing the potential for this model to contribute to enhanced energy efficiency and sustainability in both residential and commercial environments. Overall, this study underscores the transformative potential of machine learning in energy management, offering a scalable solution that can adapt to the evolving energy needs of diverse applications while promoting environmental sustainability.

## Dataset gathering

As highlighted in the introduction, we adopted supervised machine learning models in this study to automate the energy management process Fig. 1. Accordingly, the dataset gathering is an essential setup that directly contributes to the model’s effectiveness. Herein, we gathered four different datasets that express four various categories of loads studied in this manuscript. These datasets include lighting loads, industrial loads, Heating, Ventilation, and Air Conditioning (HVAC) loads, and residential loads. For various loads, we mainly utilized a combination of online available datasets from literature^9,11,12,23,31–33^. The dataset provides detailed information regarding energy usage metrics from multiple households within a smart home environment, serving as a valuable resource for researchers, data scientists, and developers interested in understanding energy consumption patterns and improving energy management systems. This dataset was collected from smart homes equipped with various sensors and devices that monitor energy consumption in real-time, enabling the analysis of how different household activities contribute to overall energy use^31,32^. The dataset includes several key features that represent different aspects of energy consumption, such as precise timestamps that record when energy usage occurs, energy consumption values indicating the amount of energy consumed by appliances and systems within the home (typically presented in kilowatt-hours or kWh), and details regarding specific appliances and their energy consumption patterns. These features allow for a granular analysis of usage habits. Additionally, the dataset provides the ability to identify target variables for various analyses, including energy usage trends over time, peak consumption periods, and comparisons between different types of appliances. All datasets are attached to this paper as Supplementary Materials 1. In general, common factors are associated with the four datasets related to the power consumption and efficiency per alternative. These two parameters are treated as the main core parameters utilized to evaluate the device inputted to the model with respect to similar alternatives in the dataset.

## Machine learning model

Following the dataset collection described in Sect. 2, we utilized a series of classification and regression machine learning models to conduct our targeted energy management model. Initially, we classify the input load within the four classifications highlighted earlier in Sect. 2. Consequently, the targeted load is embedded to be sub-categorized into one of the four sub-categories per class, from A to D. Herein, the four sub-categories are correlated to both the power consumption and efficiency per device. Consequently, the sub-category A refers to the highest power consumption-efficiency combination, while sub-category D indicates the lowest state. This process is implemented through various supervised ML algorithms (see Table 1), where the Random Forest (RF) machine learning algorithm^34–37^ was chosen based on the evaluation performance matrix, which includes model accuracy, precision, recall, and F1-score, cf. Table 1. Random Forest is a powerful ensemble machine learning algorithm that constructs a multitude of decision trees during training and outputs the mode of their predictions for classification tasks or the average for regression tasks^34–37^. One of the key advantages of using Random Forest in energy management datasets is its ability to handle large datasets with numerous features while maintaining high accuracy and robustness against overfitting. This is particularly beneficial in energy management, where datasets often contain complex and non-linear relationships among variables such as energy consumption, time of day, and appliance usage patterns. Additionally, Random Forest provides built-in feature importance measurement, allowing practitioners to identify which factors most significantly impact energy consumption, thus enabling targeted interventions. Its resilience to noise and capacity for capturing interactions among variables make it an ideal choice for analyzing energy data, enhancing the predictive power of models used for demand forecasting, load management, and energy optimization strategies.

**Fig. 2:**
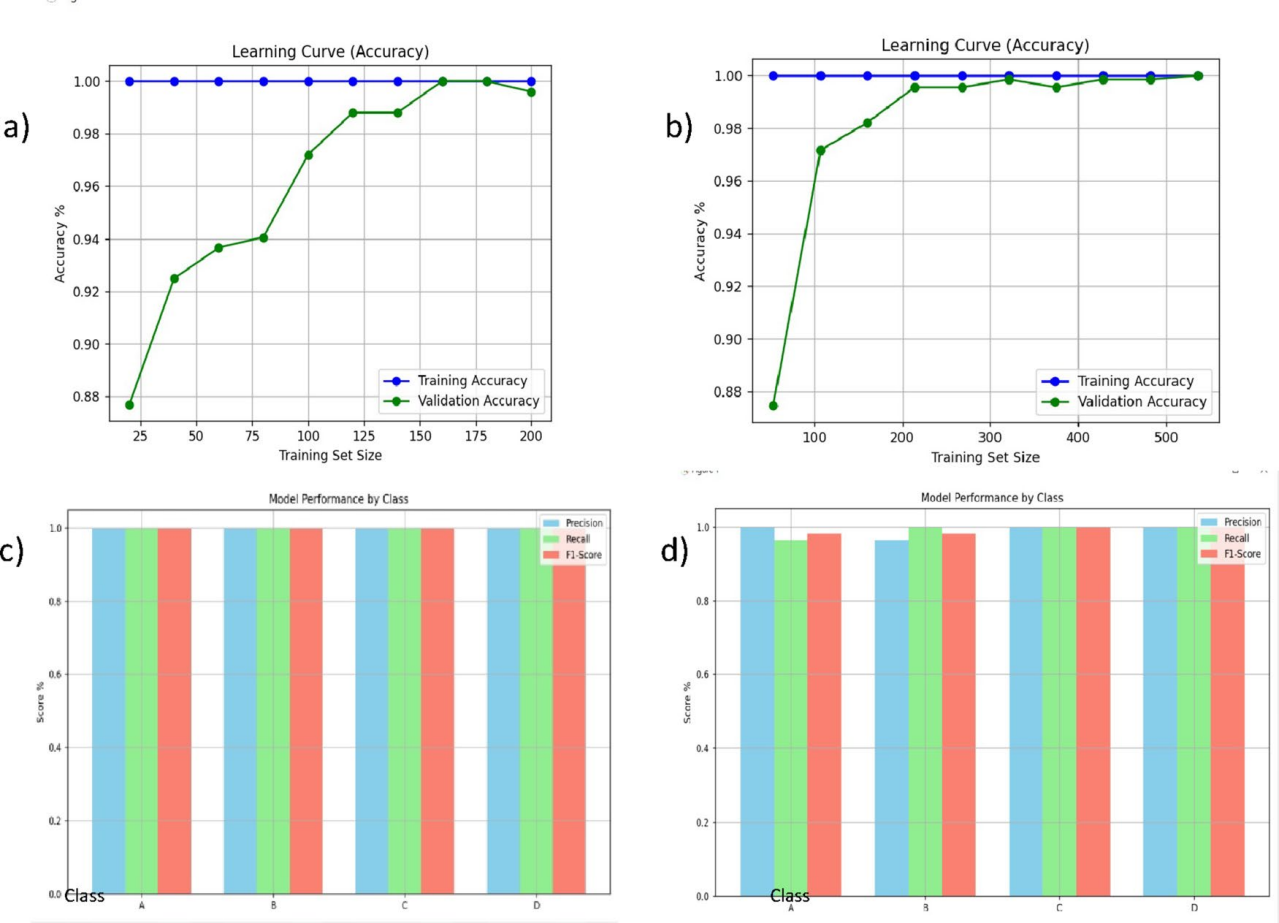
The accuracy learning curve for (**a**) the first dataset, and (**b**) the second dataset, as well as the model performance class for (**a**) the first dataset, and (**b**) the second dataset.

**Fig. 3:**
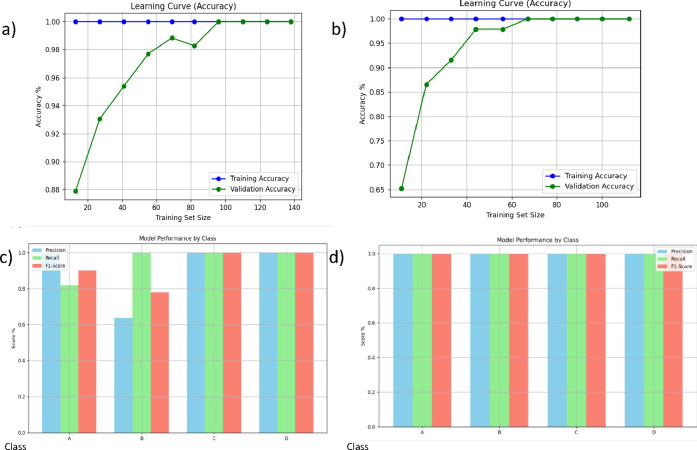
The accuracy learning curve for (**a**) the third dataset, and (**b**) the fourth dataset, as well as the model performance class for (**a**) the third dataset, and (**b**) the fourth dataset.

**Fig. 4:**
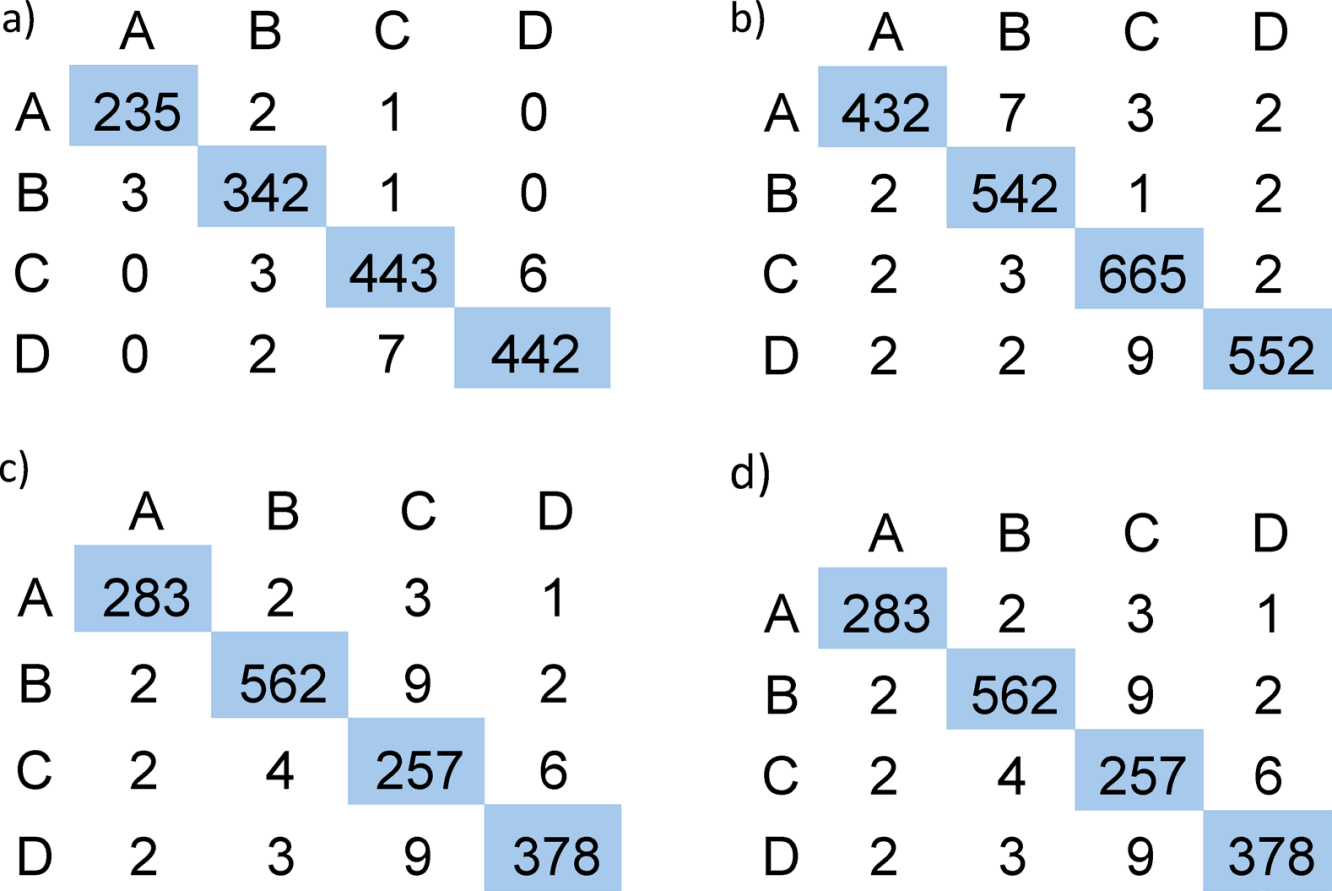
The confusion matrix for the (**a**) first dataset, (**b**) the second dataset, (**c**) the third dataset, and (**d**) the fourth dataset.

The four datasets investigated in this paper have been partitioned into 70% training, 30% validation, and testing, following the train/validation/test demonstrated in Table 2. The accuracy learning curves and the model performance by class for the four datasets are displayed in Figs. 2 and 3, while the confusion matrices are displayed in Fig. 4. Generally, the output indicators, including accuracy, precision, F1 score, and recall, showed very acceptable levels. The validation accuracy also stabilizes around 98% after reaching enough samples. Both graphs exhibit similarly high-performance levels across the four classes, with scores nearing 1.0 for precision and recall, indicating that the model performs robustly in distinguishing and classifying each category. The consistent F1 scores indicate balanced performance in terms of precision and recall for all classes. Overall, these metrics highlight the effectiveness and reliability of the model in accurately predicting outcomes in a multi-class setting. It is worth highlighting that in case the inputted device is categorized in subcategory A or B, it is automatically added to the dataset, leading to a self-learning dynamic dataset.

Regarding hyperparameter optimization, we employed two widely recognized methodologies: grid search and randomized search. The grid search technique entails the establishment of a hyperparameter value grid, followed by a comprehensive examination of all conceivable combinations to identify the optimal hyperparameter set. In contrast, randomized search selects hyperparameter values at random from a specified distribution and assesses their effectiveness based on the performance metrics derived from the data. Although hyperparameter tuning can substantially enhance the efficacy of machine learning models, it is often associated with considerable computational demands and time consumption. Nevertheless, recognizing the critical importance of attaining optimal model performance, we considered it essential to allocate substantial resources to this preliminary phase of our analysis. The entire process is illustrated in the flowchart depicted in Fig. 1. Regarding the computational expenses associated with executing the hyperparameter optimization and the machine learning models, all computations were conducted on our laboratory workstation. This computational setup features a dual Xeon Gold 6240 processor operating at 2.6 GHz, comprising 36 cores and 24 MB of cache, accompanied by 32 GB of RAM and supported by dual 480 GB SSD hard drives.

**Table 1:** The evaluation parameters associated with various machine learning algorithms across the four datasets examined in this paper.

Dataset #1				
ML model	Accuracy	Precision	recall	F1-Score
Decision Tree	88.2%	0.93	0.92	0.89
Elastic Net	76.2%	0.77	0.63	0.74
Gradient Boosting	76.2%	0.53	0.52	0.52
KNN	89.5%	0.81	0.82	0.83
Lasso	-	-	-	-
Linear Regression	-	-	-	-
Ridge Prediction	93.1%	0.91	0.91	0.87
SVM	88.3%	0.87	0.89	0.64
XG-Boost Prediction	94.2%	0.69	0.64	0.69
Random Forest	99.1%	0.92	0.96	0.93
Dataset #2				
Decision Tree	89.2%	0.91	0.89	0.87
Elastic Net	66.8%	0.78	0.65	0.64
Gradient Boosting	63.8%	0.55	0.63	0.59
KNN	92.3%	0.82	0.83	0.72
Lasso	-	-	-	-
Linear Regression	-	-	-	-
Ridge Prediction	93.1%	0.92	0.93	0.92
SVM	97.4%	0.77	0.87	0.63
XG-Boost Prediction	93.1%	0.77	0.64	0.55
Random Forest	99.7%	0.96	0.94	0.93
Dataset #3				
Decision Tree	91.2%	0.91	0.90	0.91
Elastic Net	66.8%	0.78	0.65	0.64
Gradient Boosting	56.7%	0.58	0.56	0.59
KNN	96.5%	0.84	0.83	0.82
Lasso	-	-	-	-
Linear Regression	-	-	-	-
Ridge Prediction	93.1%	0.92	0.93	0.91
SVM	79.4%	0.77	0.68	0.63
XG-Boost Prediction	93.1%	0.69	0.64	0.55
Random Forest	99.2%	0.91	0.92	0.93
Dataset #1				
Decision Tree	91.2%	0.91	0.90	0.91
Elastic Net	66.8%	0.78	0.65	0.64
Gradient Boosting	56.9%	0.51	0.56	0.52
KNN	96.5%	0.84	0.83	0.82
Lasso	-	-	-	-
Linear Regression	-	-	-	-
Ridge Prediction	80.1%	0.92	0.93	0.91
SVM	88.1%	0.77	0.68	0.63
XG-Boost Prediction	83.1%	0.79	0.62	0.82
Random Forest	99.4%	0.98	0.96	0.95

**Table 2:** The evaluation parameters associated with various training to validation, and testing schemes.

Dataset #1				
Training: validation and testing	Accuracy	Precision	recall	F1-Score
50:50	67.3%	0.61	0.60	0.61
60:40	76.8%	0.78	0.65	0.64
70:30	99.1%	0.92	0.96	0.93
80:20	96.5%	0.84	0.83	0.82
90:10	92.2%	0.78	0.77	0.79
Dataset #2				
50:50	61.2%	0.61	0.60	0.57
60:40	66.8%	0.78	0.65	0.64
70:30	99.7%	0.96	0.94	0.93
80:20	96.5%	0.84	0.91	0.92
90:10	91.9%	0.77	0.88	0.81
Dataset #3				
50:50	71.2%	0.69	0.55	0.61
60:40	76.8%	0.78	0.65	0.64
70:30	99.2%	0.91	0.92	0.93
80:20	86.2%	0.84	0.83	0.82
90:10	84.2%	0.72	0.80	0.79
Dataset #4				
50:50	78.2%	0.51	0.70	0.71
60:40	78.8%	0.72	0.78	0.84
70:30	99.4%	0.98	0.96	0.95
80:20	86.5%	0.83	0.77	0.82
90:10	82.2%	0.77	0.69	0.81

## Energy management model

Considering the trained ML model discussed in Sect. 3, this section illustrates the entire mathematical model used to determine the energy saving with respect to the reference status. Principally, each device, either from the running model or the suggested model, has a total energy consumption:

1 $$\:E=P\:\:T$$

where $\:P$ is power and $\:T$ is time in hours per day. In the benchmarking phase, the device is assessed to be in sub-category A to D based on the assessment of both power and efficiency with respect to the correlated dataset. Herein, a device is automatically replaced by another device from the dataset list, with the same technical specification, if the device is subcategorized in subcategory D. For subcategory C, a warning and recordation for smarting is outputted, while for subcategories A and B, no action is taken. Referring to those devices in subcategory D, while still assuming the same number of operating hours, the energy saving per device can be expressed by:

2 $$\:{E}_{saving\:}=\left({P}_{running}-{P}_{alternative}\right)T\:\:$$

where $\:{P}_{running},\:$ and$\:{P}_{alternative}$ are the power of the running device and the alternative device, respectively. Although the value of $\:{E}_{saving\:}$is positive all the time, as far as the device is categorized as a sub-category D device, the action is also correlated to the economic part of the decision. In other words, the capital cost of the alternative device should be taken into consideration. The payback period ($\:{N}_{payback\:}$) for the alternative device is estimated from:

$$\:{C}_{CC,alternative\:}\:\:{C}_{CC\:}-$$

3 $$\sum\:_{n=1}^{{N}_{payback\:}}{E}_{saving\:}\:{C}_{ET}\:\:\:{C}_{ET\:}\:365\:(1-i\:\:\:i{)}^{n\:}-{C}_{salvage\:value,running\:}=0$$

where $\:{C}_{CC,alternative}$ is the capital cost of the alternative device in LE., $\:{C}_{ET}$ is the electricity tariff level based on the unit consumption in LE., $\:i$ is the inflation rate, and $\:{C}_{salvage\:value,running\:}$is the salvage value of the running device. Herein, we adopted the electricity tariff approved by the Egyptian government in July 2024^38^, and the Egyptian Central Bank announced the inflation rate^39,40^. The term is introduced to represent the uncertainty factor, making it capable of conducting a sensitivity analysis to address the impact of capital cost, tariff, and inflation rate on the model decision-making processes. Accordingly, the alternation action is implemented if and only if:

4 $$\:{N}_{payback\:}<\:\:{(N}_{lifetime\:}\:{N}_{lifetime\:})\:$$

where is an empirical coefficient based on the nature of the device with a maximum value of 1, and a minimum value of 0.65, while$\:{N}_{lifetime\:}$, and $\:{N}_{lifetime\:}$are the alternative device lifetime, and uncertainty changes, respectively.

In sequence, the benchmarking phase is followed by the smarting phase. The main objective of this phase is to optimize the working hours of the device, seeking minimum energy consumption. Accordingly, the algorithm suggests a smart time management sensor to be added to minimize the energy as follows:

5 $$\:{E}_{smarting}=P\:\:({T}_{running\:}-{T}_{smarting})\:$$

where $\:{T}_{running\:}$is the current operating time of the running device, while $\:{T}_{smarting}$ is the optimized operating time after adding sensors. Again, the $\:{E}_{smarting}$ will show a positive value all the time; however, the cost of smarting still needs to be investigated. Consequently, we explored the economic feasibility of the smarting phase through the equation:

$$\:{C}_{CC,smarting\:\:}\:\:{C}_{CC\:}-$$

6 $$\sum\:_{k=1}^{{N}_{smmarting\:\:}}{E}_{smarting\:}\:{C}_{ET}\:\:{C}_{ET\:}\:365\:(1-i\:\:i{)}^{k}=0$$

where $\:{C}_{CC,smarting\:}$ is the entire capital cost associated with the smarting phase in LE. Accordingly, the smarting action is implemented if and only if:

7 $$\:{N}_{smmarting\:\:}<\:\:{N}_{smmarting,\:lifetime\:}\:\:{N}_{smmarting,\:lifetime\:}$$

where $\:{N}_{smmarting,\:lifetime\:}$is the sensor’s lifetime. These equations are all scripted through Python as a post-processing stage to the ML classification algorithms, resulting in a final estimation for the entire energy saving across the whole system by iterating the script over all the inputted devices.

## Case studies

**Fig. 5:**
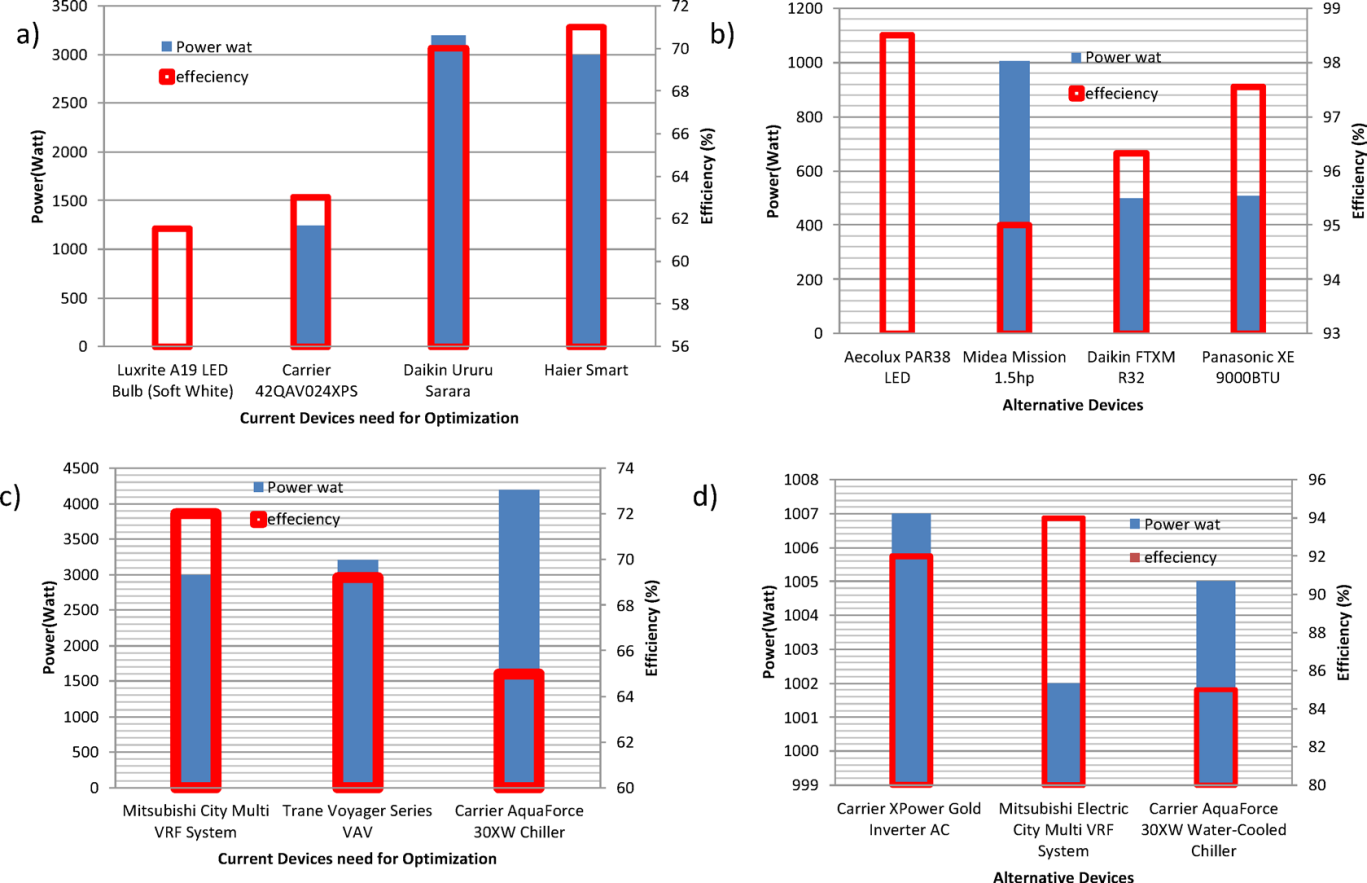
The power-efficiency data for both the (**a**), (**c**) running and the (**b**), (**d**) alternative devices for case studies #, and #2, respectively.

**Fig. 6:**
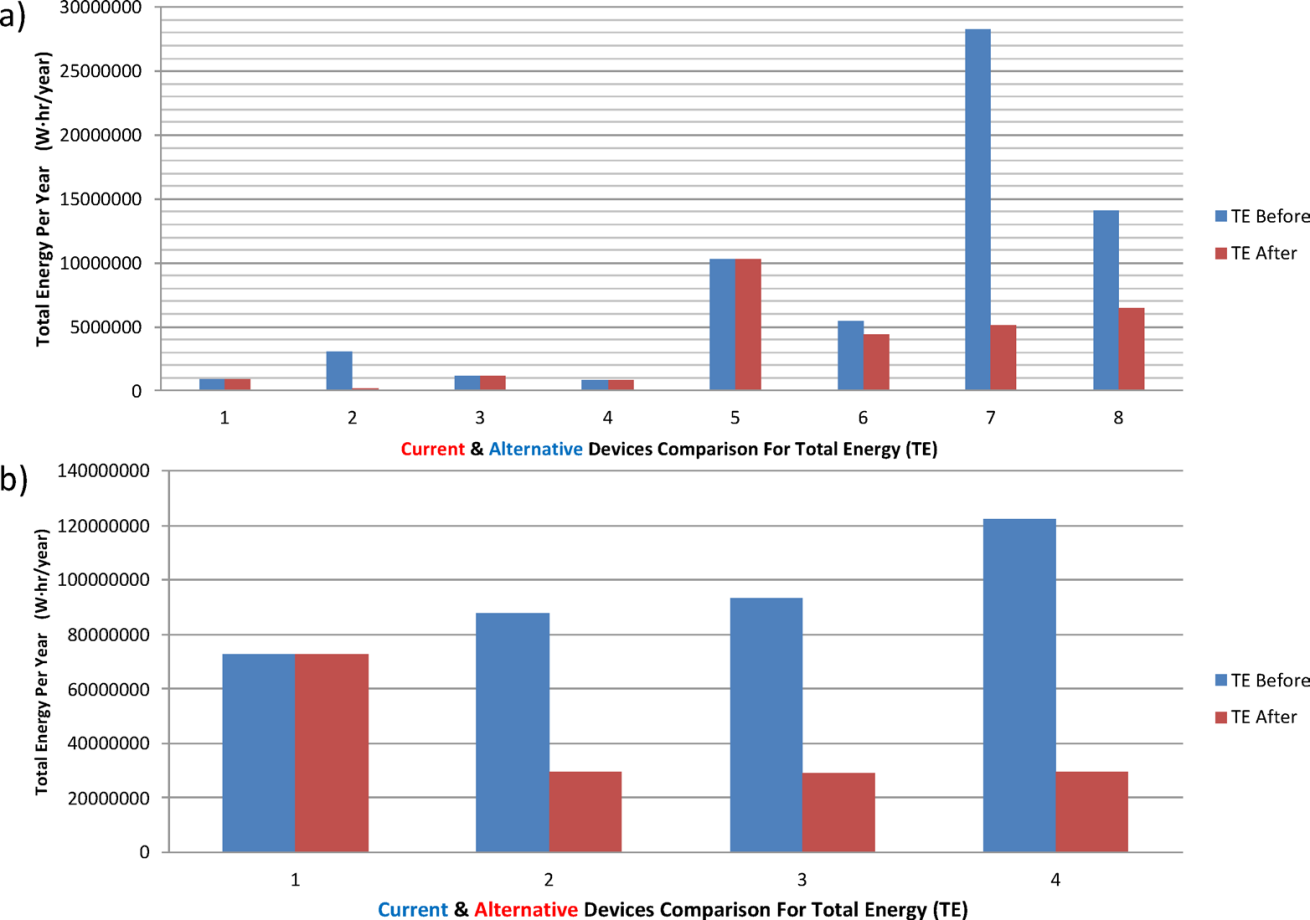
The total energy before and after AI for the case studies (**a**) #1, and (**b**) #2.

The developed model has been tested among four different case studies to assess its capability to manage the energy consumption in a techno-economic manner. The process as described in the flow chart in Fig. 1 is initiated by importing the CSV file that includes the running loads to be optimized. Consequently, each row from the imported file is processed through our three cascaded stages: load classification, benchmarking, and smarting. In sequence, the system will be outputted with an optimized CSV file, which is a combination of alternative loads, or running loads, in case action is recommended to a specific input. In addition, the model can determine the expected energy and cost savings. The first selected case study, with a sample of input data in Table 3, represents a typical essential load in Egypt. Applying the procedure in Fig. 1, four items of the eight presented items have been alternated with lower energy consumption alternatives. The power-efficiency data (Fig. 5) for both the running and the alternative devices are displayed in Figs. 5-a and b, see Table 4. In addition, the energy consumption of the system is demonstrated in Fig. 6-a, before and after applying the model, indicating either energy-saving action or no action as described. The model showed an overall energy saving of 34.73 MWh/year with an overall cost saving of 78,145.12 LE/year.

**Table 3:** Running loads for case study #1.

#	Name	Type	Qty	Power (W)	Time (Hrs)	Efficiency
1	LED Bulb (Soft White)	light	35	5	14	72
2	LED light fixture IP44	light	40	15	14	61
3	CS/CU-XU18XKY	TV	3	127	8	80
4	Q80A	TV	3	111	7	91
5	AC	AC	3	1569	6	86
6	42QAV024XPS	AC	2	1245	6	63
7	HVAC	HVAC	4	3200	7	70
8	HVAC	HVAC	5	3000	7	71

**Table 4:** Updated loads after adding alternative loads for case study #1.

#	Name	Type	Qty	Power (W)	Time (Hrs)	Efficiency
1	LED Bulb (Soft White)	light	35	5	14	72
2	PAR38 LED	light	40	1	14	98.5
3	CS/CU-XU18XKY	TV	3	127	8	80
4	Q80A	TV	3	111	7	91
5	AC	AC	3	1569	6	86
6	AC 1.5hp	AC	2	1005	6	95
7	FTXM R32	HVAC	4	500	7	96.33
8	XE 9000BTU	HVAC	5	509	7	97.55

**Fig. 7:**
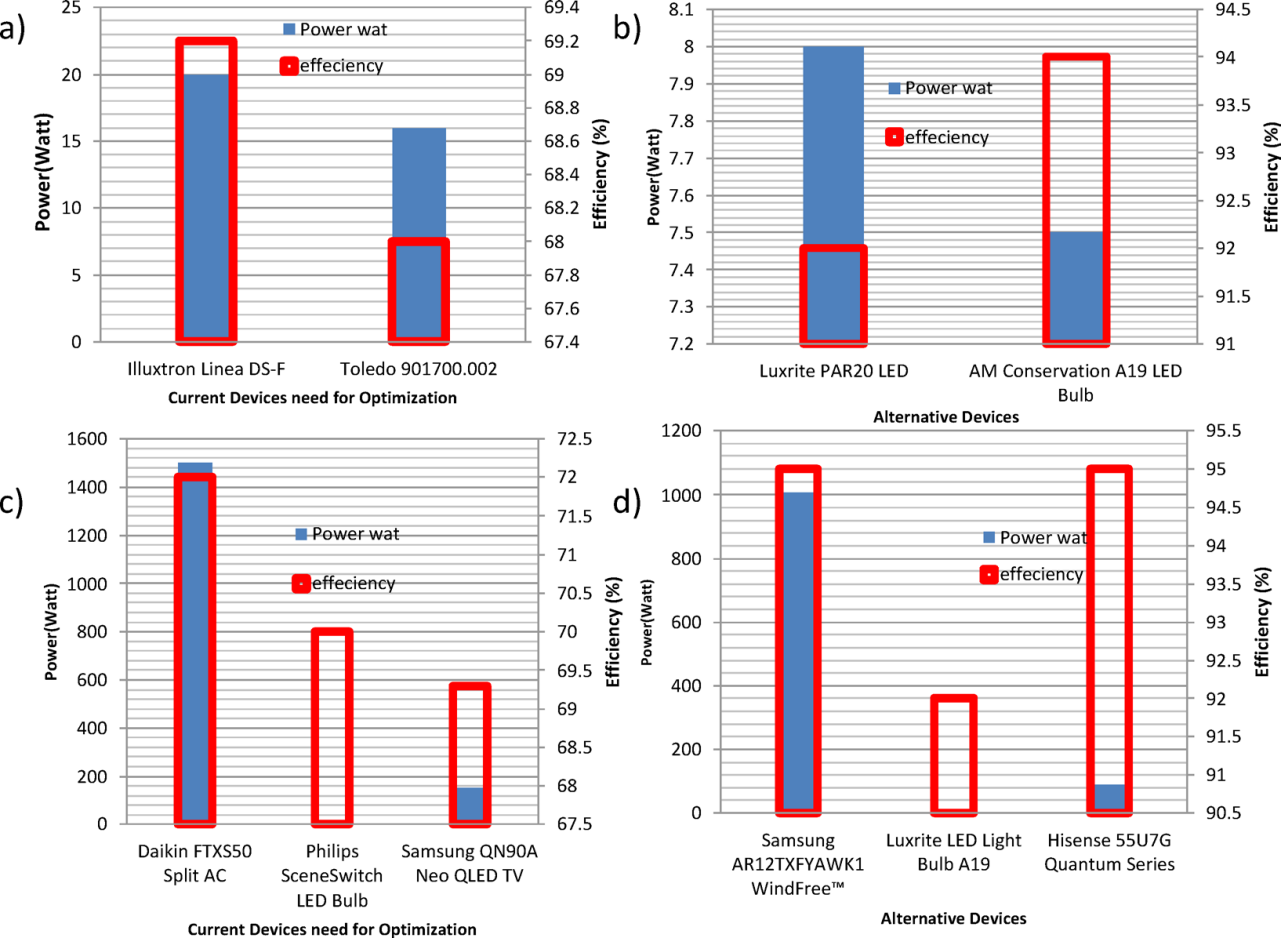
The power-efficiency data for both the (**a**), (**c**) running and the (**b**), (**d**) alternative devices for case studies #3, and #4, respectively.

**Fig. 8:**
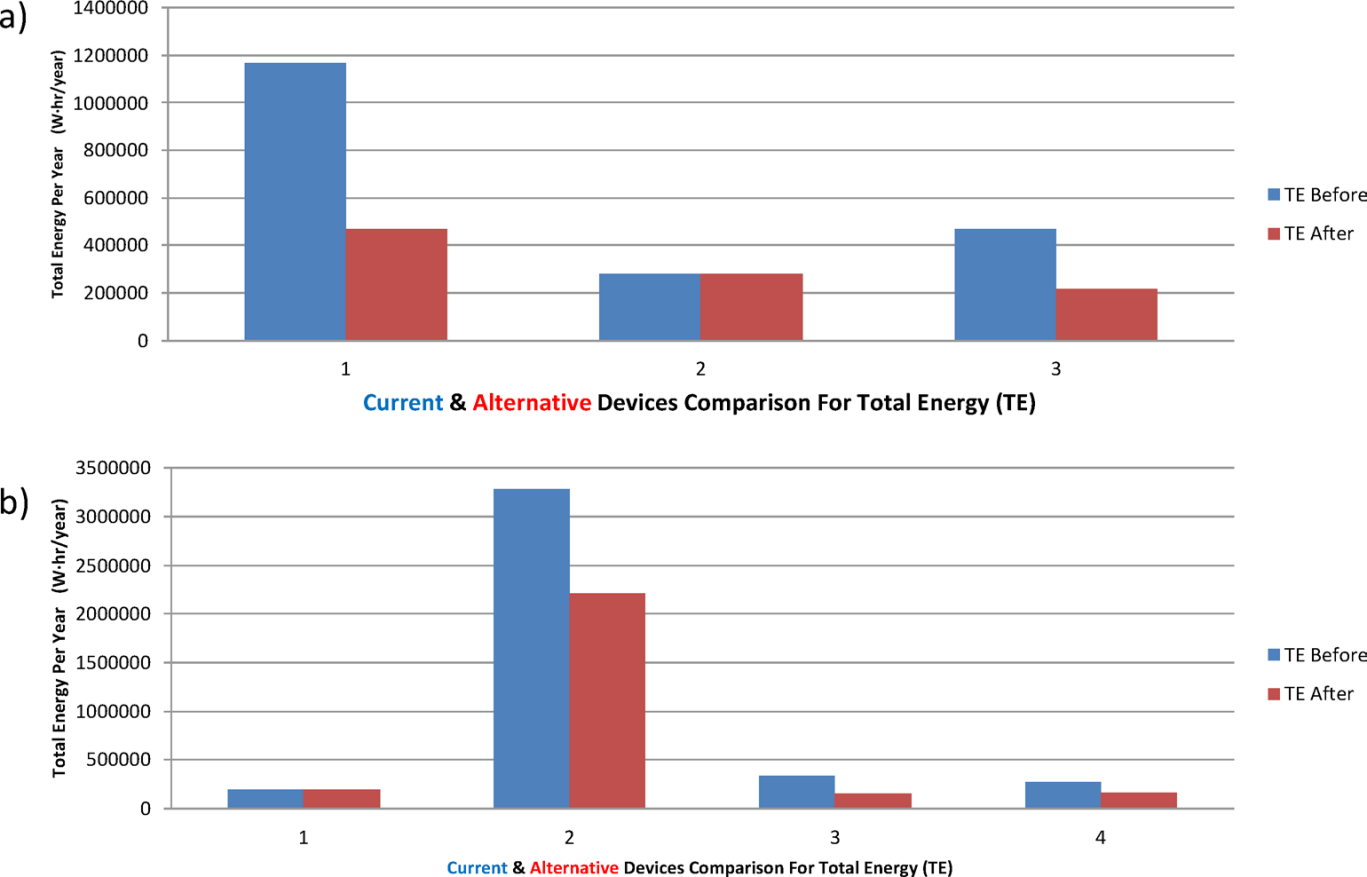
The total energy before and after the AI model for the case studies (**a**) #3, and (**b**) #4.

The second case study was focused on HVAC systems in a university building, as listed in Table 5. Three of the presented four items have been alternated with lower energy consumption alternatives. The power-efficiency data for both the running and the alternative devices are displayed in Figs. 5-c, and d, with the optimized list in Table 6. In addition, the energy consumption of the system is demonstrated in Figs. 6-a, b, before and after applying the model, indicating either energy saving action or no action as described. The model showed an overall energy saving of 215.67 MWh/year with an overall cost saving of 463,693.08 LE/year. The third case study described a hybrid lighting system implementation in a bank branch within a shopping mall. The running loads are listed in Table 7. The presented list has been alternated with lower energy consumption alternatives, see Table 8. The power-efficiency data (Fig. 7) for both the running and the alternative devices are displayed in Figs. 7-a, and b. In addition, the energy consumption of the system is demonstrated in Fig. 8-a, before and after applying the model, indicating either energy saving action or no action as described. The model showed an overall energy saving of 0.9 MWh/year with an overall cost saving of 2,040.35 LE/year. Finally, a Europium residential house is treated as our case study number 4, see Table 9. The power-efficiency data for both the running and the alternative devices are displayed in Figs. 7-c, and d. In addition, the energy consumption of the system is demonstrated in Fig. 8-b, before and after applying the model, indicating either energy saving action or no action as described. The model showed an overall energy saving of 0.9 MWh/year with an overall cost saving of 2,040.35 LE/year, see Table 10. Through observing all the document results, it can be concluded that the model verified its reliability and effectiveness under various types of loads. In addition, the model has an expandable feature to enable more databases for different types of loads. Such additional features can open the door for extensive research toward a sufficient AI-based energy auditing and management tool.

The results presented in this section were derived under the assumption of zero uncertainty. However, to gain a more nuanced understanding of the model’s robustness, we conducted a sensitivity analysis utilizing t factor introduced in Sect. 4. This analysis serves to identify critical turning points for each sensitivity factor that could significantly influence the model’s decision-making process. Specifically, for the uncertainty associated with capital costs $\:\:{C}_{CC\:}$, we estimated a sensitivity of 4.7%. This indicates that variations in capital costs within this range could lead to noticeable shifts in the model’s outcomes. Furthermore, the analysis revealed that fluctuations in the electricity tariff $\:\:{C}_{ET}$ could reach as high as 9.7% without adversely impacting the system’s overall decision. Additionally, the uncertainty related to the inflation rate was found to be 11.5%, suggesting that changes in inflation could have a substantial effect on the economic viability of the model. Lastly, the potential variation in the expected lifetime of the assets was estimated to approach 10.6%, indicating that uncertainties in asset longevity could also play a significant role in the model’s performance. Overall, these sensitivity analysis results highlight the importance of considering various uncertainties in economic modeling, as they can have significant implications for decision-making and strategic planning. By identifying these critical factors, stakeholders can better prepare for potential risks and make more informed choices regarding investments and operational strategies.

While this study presents a robust framework for autonomous energy management and auditing, several limitations warrant consideration, particularly as part of future extensions. One significant challenge is sensor calibration, as accurate energy monitoring relies on the precise functioning of sensors, which may drift over time or be affected by environmental factors. Ensuring consistent calibration protocols is essential to maintain data accuracy. Additionally, data quality assurance is critical; the effectiveness of the machine learning algorithms hinges on the integrity of the input data. Inaccurate or inconsistent data can lead to suboptimal performance and misinformed decision-making. Privacy and cybersecurity are also paramount concerns, especially given the reliance on IoT devices for real-time data streaming. Safeguarding sensitive information and ensuring secure communication channels are vital to protect against potential breaches. Furthermore, the system must effectively handle missing or anomalous data in real-time to maintain operational reliability. Implementing robust data imputation techniques and anomaly detection algorithms will be crucial in addressing these issues, ensuring that the model remains resilient and effective under varying conditions. Addressing these limitations will enhance the model’s applicability and reliability, paving the way for more comprehensive energy management solutions in the future.

**Table 5:** Running loads for case study #2.

#	Name	Type	Qty	Power (W)	Time (Hrs)	Efficiency
1	Split Type AC	HVAC	10	2500	8	76
2	VRF System	HVAC	10	3000	8	72
3	VAV	HVAC	10	3200	8	69.2
4	AquaForce 30XW Chiller	HVAC	10	4200	8	65

**Table 6:** Updated loads after adding alternative loads for case study #2.

#	Name	Type	Qty	Power (W)	Time (Hrs)	Efficiency
1	Split Type AC	HVAC	10	2500	8	76
2	XPower Gold Inverter AC	HVAC	10	1007	8	92
3	Electric City Multi VRF System	HVAC	10	1002	8	94
4	30XW Water-Cooled Chiller	HVAC	10	1005	8	85

**Table 7:** Running loads for case study #3.

#	Name	Type	Qty	Power (W)	Time (Hrs)	Efficiency
1	Linea DS-F	light	20	2500	20	69.2
2	NEONICA LED Strip 3528	light	10	3000	9.6	75
3	901700.002	light	10	3200	16	68

**Table 8:** Updated loads after adding alternative loads for case study #3.

#	Name	Type	Qty	Power (W)	Time (Hrs)	Efficiency
1	FTKM Series – Split Type AC	AC	10	2500	8	92
2	XPower Gold Inverter AC	AC	10	1007	8	94
3	Electric City Multi VRF System	AC	10	1002	8	68

**Table 9:** Running loads for case study #4.

#	Name	Type	Qty	Power (W)	Time (Hrs)	Efficiency
1	LG OLED55C2 Television	Residential	1	110	5	90
2	FTXS50 Split AC	HVAC	1	1500	6	72
3	SceneSwitch LED Bulb	Light	8	13	9	70
4	QN90A Neo QLED TV	Residential	1	150	5	69.3

**Table 10:** Updated loads after adding alternative loads for case study #4.

#	Name	Type	Qty	Power (W)	Time (Hrs)	Efficiency
1	OLED55C2 Television	Residential	1	110	5	90
2	AR12TXFYAWK1 WindFree™	HVAC	1	1007	6	95
3	LED Light Bulb A19	Light	8	6	9	92
4	55U7G Quantum Series	Residential	1	90	5	95

## Conclusion

In conclusion, this study demonstrates the significant potential of implementing a fully automated energy management and auditing system harnessing machine learning techniques to enhance energy efficiency across various applications. The three-phase model—comprising load classification, benchmarking, and smart monitoring—enables precise categorization of energy loads, establishment of optimal performance standards, and continuous real-time tracking of energy consumption. The successful application of this model in diverse case studies not only validates its effectiveness in achieving substantial energy savings and cost reductions but also highlights its adaptability to different environments and operational contexts. By promoting smarter energy management practices, this framework contributes to the advancement of sustainability initiatives, underscoring the critical role of innovative technologies in addressing the growing global demand for energy efficiency and environmental stewardship.

The scalability of the proposed autonomous energy management model extends beyond the immediate context of the case studies presented, offering significant potential for adaptation in diverse geographical regions and varying energy markets. By leveraging machine learning techniques that can be tailored to local energy consumption patterns and regulatory frameworks, the model can effectively address the unique challenges faced by different regions. For instance, in areas with high renewable energy penetration, the model can optimize energy usage by integrating real-time data from solar or wind generation sources, thereby enhancing grid stability and efficiency. Furthermore, the framework’s flexibility allows for the incorporation of region-specific benchmarks and performance standards, ensuring that energy management strategies remain relevant and effective across different contexts. As such, this model not only promotes energy efficiency and sustainability within the original study parameters but also serves as a scalable solution that can be deployed in various global settings, ultimately contributing to a more resilient and sustainable energy landscape worldwide.

## Supplementary Information

Below is the link to the electronic supplementary material.

Supplementary Material 1

Supplementary Material 2

Supplementary Material 3

Supplementary Material 4

## Data Availability

The data supporting this study’s findings are available from the corresponding author upon reasonable request.
